# Retraction Note: Upregulation of the long non-coding RNA AFAP1-AS1 affects the proliferation, invasion and survival of tongue squamous cell carcinoma via the Wnt/β-catenin signaling pathway

**DOI:** 10.1186/s12943-019-1026-y

**Published:** 2019-06-01

**Authors:** Ze-you Wang, Min Hu, Min-hui Dai, Jing Xiong, Shuai Zhang, Han-jiang Wu, Shan-shan Zhang, Zhao-jian Gong

**Affiliations:** 1 Department of Laboratory Medicine, The Second Xiangya Hospital, Central South University, Changsha, 410011 Hunan China; 2 Department of Ophthalmology, Xiangya Hospital, Central South University, Changsha, 410008 Hunan China; 3 Department of Clinical Medicine, Fujian Medical University, Fuzhou, 350100 Fujian China; 4 Department of Oral and Maxillofacial Surgery, The Second Xiangya Hospital, Central South University, Changsha, 410011 Hunan China; 5 Department of Stomatology, Xiangya Hospital, Central South University, Changsha, 410008 Hunan China

**Retraction note to:** **Molecular Cancer**

**https://doi.org/10.1186/s12943-017-0752-2**

The authors have retracted this article [1] because there are errors in Figures 4a and 4b. In these figures a number of cell field views are replicated between experimental groups:

In Figure 4b the Scc-9/Mock panel replicates the Scc-9/shR-NC panel in Figure 4a

In Figure 4b the CAL-27/Mock panel replicates the CAL-27/Mock panel in Figure 4a

In Figure 4b the CAL-27/sHR-Nc panel replicates the CAL-27/sHR-Nc panel in Figure 4a

In Figure 4b the CAL-27/shR-AFAP1-AS1 panel replicates the CAL-27/shR-AFAP1-AS1 panel in Figure 4a

The conclusions of this study are therefore not reliable. Zhaojian Gong, Hanjiang Wu, Shanshan Zhang, and Shuai Zhang agree with this retraction. Ze-you Wang, Min Hu, Min-hui Dai, and Jing Xiong agree with the retraction but did not respond to further correspondence about the retraction notice.
